# Academic vs. industry-sponsored trials: A global survey on differences, similarities, and future improvements

**DOI:** 10.7189/jogh.14.04204

**Published:** 2024-11-22

**Authors:** Jean-Marc Hoffmann, Annina Bauer, Regina Grossmann

**Affiliations:** 1 Clinical Trials Center, University of Zurich/University Hospital Zurich, Zurich, Switzerland; 2 Institute of Biomedical Ethics and History of Medicine (IBME), University of Zurich, Zurich, Switzerland

## Abstract

**Background:**

Clinical research is marked by its multifaceted nature, presenting a multitude of different approaches, designs, and objectives that can complicate the planning, initiation, and conduct of clinical trials. The role and organisation of the sponsor institution are pivotal in this context. We aimed to investigate possible challenges and needs, including their underlying factors, for academia and industry during the set-up and conduct of clinical trials.

**Methods:**

We conducted a cross-sectional survey-based study within an international network of highly qualified academic research institutions (ARIs). The main outcome measures were the regulatory framework for clinical trials, scope and organisation of academic and industry-sponsored trials, funding sources of academic clinical trials, submission and approval process, as well as study conduct of academic vs. industry-sponsored trials.

**Results:**

We surveyed employees of ARIs with extensive experience in phase I–IV clinical trials. All ARIs participated in academic clinical trials and 90% were involved in industry-sponsored trials. Respondents reported that academic trials faced greater challenges in communication with relevant institutional review boards/ethics committees and competent authorities compared to industry-sponsored trials. Additionally, academic trials were found to have significantly less financial support during their conduct. Specific challenges for academia vs. industry included ‘insufficient personnel resources’ (60% vs. 50%), ‘recruitment problems’ (60% vs. 78%) and ‘lack of knowledge/experience’ (35% vs. 11%).

**Conclusions:**

Our findings indicate that industry-sponsored trials encounter fewer issues in set-up, funding, and trial conduct compared to academic trials. Improving collaboration between academic sponsors and ARIs is essential to address these challenges. ARIs provide critical support and guidance for academic researchers, not only in planning and implementing projects, but also in assessing feasibility and securing funding.

Clinical research is inherently complex, involving diverse approaches, designs, and objectives that can complicate trial planning, initiation, and conduct. The sponsor institution's role and organisation are pivotal in this context, significantly impacting a trial's success by addressing scientific, ethical, economic, operational, and regulatory challenges. Notably, significant differences exist between industry-sponsored trials (ISTs) and academic clinical trials (ACTs) due to their distinct clinical focusses and objectives [1–4]. By definition, ACTs are studies planned and managed within an academic context, often led by physician-researchers who also take on the sponsor role according to the International Council for Harmonisation of Technical Requirements for Pharmaceuticals for Human Use Good Clinical Practice (ICH GCP) guidelines [5]. These trials are primarily funded through grants, institutional resources, and public funds, necessitating substantial time and effort from researchers. In contrast, ISTs are financed by pharmaceutical or biotechnology companies or private not-for-profit agencies, which assume overall responsibility for the trial without direct involvement in its conduct. They typically prioritise commercial interests, particularly those of pharmaceutical companies aiming to develop and gain approval for medical treatments [6].

A significant distinction between ACTs and ISTs lies in their financial capacities, with ACTs frequently facing financial constraints [1,3]. These budget limitations often result in severely restricted resources for initiating and conducting trials, sometimes leading to premature termination [1,3,4].

Further studies examining disparities between ACTs and ISTs have identified variations in their impact on clinical practice [2], research outcomes and quality [7], funding [1], and publication bias [2,8]. This highlights several ethical considerations aimed at protecting participants' interests and preserving research integrity, including prioritising participant welfare, ensuring informed consent, fostering trust and transparency, preserving data integrity, promoting equity, offering follow-up care, facilitating clear communication, maintaining scientific and social value, and fulfilling responsibilities to sponsors and funders.

Academic research institutions (ARIs), who are in the context of clinical research also known as clinical trial units or clinical trial centres, play a crucial role as centres of expertise and support, facilitating the efficient and ethical conduct of clinical trials for both ACTs and ISTs. They encounter daily challenges and difficulties that underscore the differences between these types of trials. Despite their critical role, the scientific literature lacks in exploration of the differences between ACTs and ISTs from the perspective of ARIs.

Recognising the vital role of ARIs and emphasising key ethical considerations, we aimed to explore the differences between ACTs and ISTs and the underlying factors from the viewpoint of ARIs.

## METHODS

### Study design

We designed a cross-sectional survey-based study, which was distributed within the International Clinical Trial Center Network (ICN), an international network of highly-qualified ARIs. As we did not use any personal data from survey respondents, our study did not fall under the Swiss Human Research Act and therefore did not require ethics approval or informed consent from survey participants.

### Survey conception

Our primary aim with this survey was to gain a comprehensive understanding of the processes associated with clinical trials within ARIs and the relevant regulatory frameworks. We designed the survey following a prior literature review on PubMed using the following terms: “academic clinical trial*”, “investigator initiated trial*”, “IIT”, and “industry sponsored trial*”. The survey comprised mainly close-ended questions with Likert-type response options where appropriate, including an ‘I don’t know’ option to facilitate quantitative analysis. We also included a free text section to allow participants to provide further insights.

The survey addressed several areas inadequately covered in the literature, based on our experience with ARIs: the regulatory framework for clinical trials; the scope and organisation of ACTs and ISTs; funding of ACTs; the submission process; and the study conduct of ACTs vs. ISTs. The survey was reviewed by two experts of ARIs (Freiburg, Germany and Zurich, Switzerland) with extensive knowledge in clinical trial regulation, as well as one physician-investigator and one master’s student. Their feedback was used to refine the questions, which were then validated in a final review.

The finalised version of the survey (**Online Supplementary Document**) consisted of 35 questions in English, including a section on greenhouse gas emissions of clinical trials (analysed separately [9]) and ethical aspects of ACTs. It was implemented on the REDCap platform [10], which allowed for modular question composition.

### Survey population and conduct

We targeted representatives from ARIs who were members of the ICN and had long-term experience in academic clinical research, as well as knowledge and understanding of the local context. On 18 October 2022, we sent invitation emails with instructions and a link to the REDCap survey to these representatives, seeking feedback on the conduct of ACTs vs. ISTs in their institution and their respective countries/jurisdictions or regions.

To ensure a homogeneous, GCP-compliant international sample with extensive clinical research knowledge, we sent the survey to the contact person of all ICN member institutions (i.e. steering board members, regular members, and affiliated members). In 2022, the ICN comprised 25 members. Membership criteria include adherence to GCP-compliant regulations, contribution of added value to the network, and support for clinical research activities of the associated institution/organisation [11]. The survey was open for responses from 18 October 2022 to 30 January 2023. We sent the reminders on 16 November 2022, 20 December 2022, and 10 January 2023.

### Data management and analysis

Due to the small sample size and the descriptive nature of the survey, we primarily used descriptive statistics to report our findings, assessing statistical significance between ACTs and ISTs using Fisher exact test. The analysis was performed on a per-question level where appropriate, using the general case of larger m × n contingency tables, where m (ACTs vs. ISTs) = 2 and n (answer possibilities) > 2. We did not compare individual answers between the two groups, nor did we make adjustments for multiple comparisons. We applied the CROSS reporting guideline for the reporting our findings [12].

We conducted the quantitative analysis with Microsoft Excel, version 16 (Microsoft Corporation, Redmond, Washington, USA).

## RESULTS

We analysed 20 responses out of the 20 received. As the ICN consisted of 25 members in 2022, this amounted to a response rate of 80%. Responding ARIs were from 15 different countries/jurisdictions (**Table 1**).

**Table 1:** Characteristics of the participating ARIs

Country (continent)	Institution
Nigeria (Africa)	University College Hospital
China (Asia)	Shanghai Clinical Research Center
Hong Kong (Asia)	University of Hong Kong
Israel (Asia)	Sheba Medical Center Hospital
Japan (Asia)	Kyoto University
Singapore (Asia)	Singapore Clinical Research Institute
Taiwan (Asia)	Hualien Tzu Chi Hospital, Changhua Christian Hospital, China Medical University Hospital
Thailand (Asia)	Khon Kaen University
Turkey (Asia/Europe)	Istanbul University
Australia (Australia)	University of South Australia
Austria (Europe)	University of Graz
Germany (Europe)	Technical University of Munich, Hannover Medical School, University of Freiburg, University of Duisburg-Essen
Italy (Europe)	Alessandria Hospital
Switzerland (Europe)	University Hospital Zurich
United Kingdom (Europe)	Cambridge University

ARI – academic research institution

**Figure 1:**
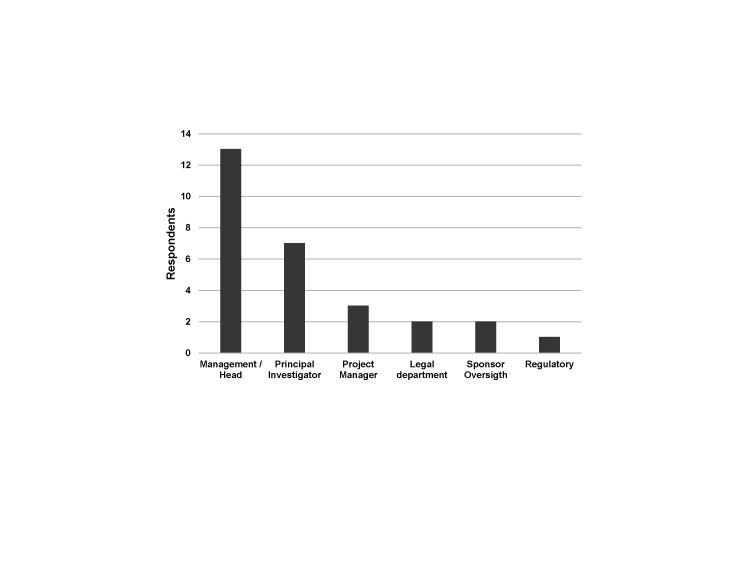
Main function/department of respondents.

Most respondents came from Europe and Asia (n = 18, 90%). More than half (n = 13, 65%) worked in a management/leading position within the ARI (**Figure 1**). All ARIs (n = 20, 100%) applied the ICH GCP guideline for their clinical trials, with the guideline being linked to their national legal text or regulation [5]. Almost all ARIs (n = 19, 95%) had a specific national law for clinical trials. Moreover, 14 (70%) of the respondents had additional local/institutional standards or guidelines, five (25%) did not, and one (5%) did not know. Most of the responding ARIs (n = 17, 85%) were involved in Phase I, 19 (95%) in Phase II and Phase III, and 18 (90%) in Phase IV clinical trials. All of the ARIs (n = 20, 100%) were involved in ACTs and 18 (90%) in ISTs. Non-clinical trials, i.e. any human research projects with the exception of clinical trials (i.e. sampling of biological material or collection of health-related personal data), were supported by 12 respondents (60%).

**Figure 2:**
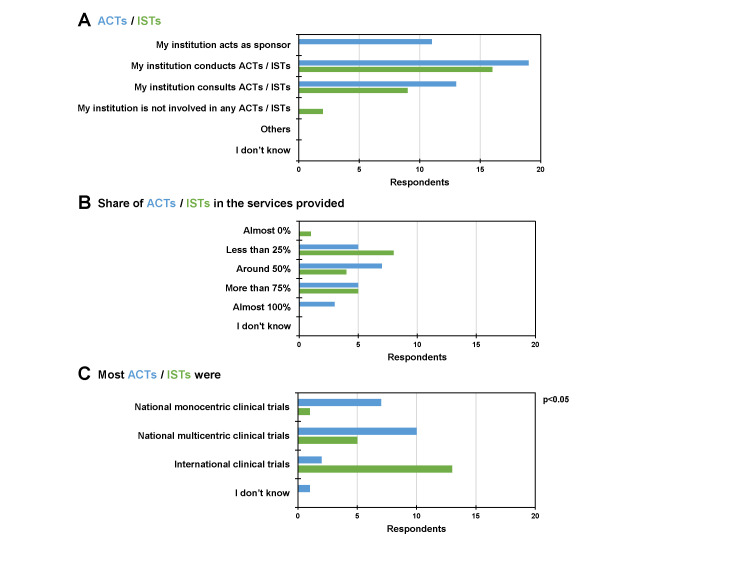
ARI involvement for ACTs and ISTs. **Panel A.** General information. **Panel B.** Share of ACTs / ISTs in the services provided. **Panel C.** Clinical trial design. ACT – academic clinical trial, ARI – academic research institution, IST – industry-sponsored trial.

We further characterised the ARIs’ involvement in clinical trials (**Figure 2**). Concerning ACTs, 11 (55%) acted as sponsors, while 19 (95%) conducted and 13 (65%) consulted these research projects. Among ISTs, 16 (80%) conducted and nine (45%) consulted them, while two (10%) were not involved in ISTs (**Figure 2**, Panel A). For the majority of ARIs (n = 15, 75%) ACTs made up a share in the services provided of 50% or more (**Figure 2**, Panel B). In contrast, ISTs only represented a share of less than 25% for over half of the ARIs (n = 11, 55%). According to half of the ARIs (n = 10, 50%), most ACTs were national multicentric clinical trials, while most ISTs were international clinical trials, according to 13 (72%) respondents (**Figure 2**, Panel C). Fisher exact test showed a significant difference in the setup of clinical trials (monocentric, multicentric, international) for ACTs vs. ISTs (*P* < 0.05).

**Figure 3:**
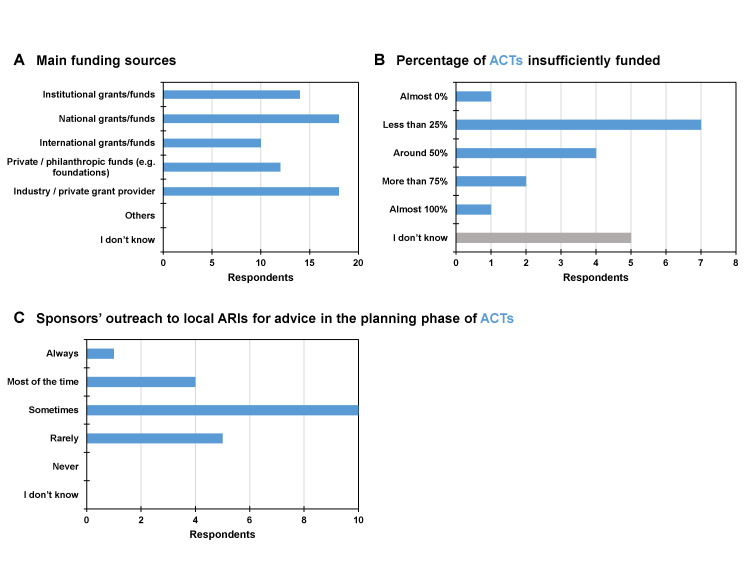
Funding and support of ACTs. **Panel A.** Main funding sources. **Panel B.** Percentage of ACTs insufficiently funded. **Panel C.** Sponsors’ outreach to local ARIs for advice in the planning phase of ACTs. ACT – academic clinical trial.

We also collected information on the funding of ACTs and, according to the respondents, the main funding sources were national grants/funds, industry/private grant providers and institutional grants/funds (**Figure 3**, Panel A). Seven (35%) respondents indicated that less than 25% of ACTs were insufficiently funded. Meanwhile, seven (35%) rated this percentage as being above 25%, with four (20%) rating it at around 50%, two (10%) at more than 75%, and one (5%) at almost 100% (**Figure 3**, Panel B). However, five respondents (25%) reported not knowing this information. Ten (50%) participants stated that sponsors sometimes reach out to local ARIs for advice in the planning phase of ACTs, while only one respondent (5%) indicated that this is always the case (**Figure 3**, Panel C).

**Figure 4:**
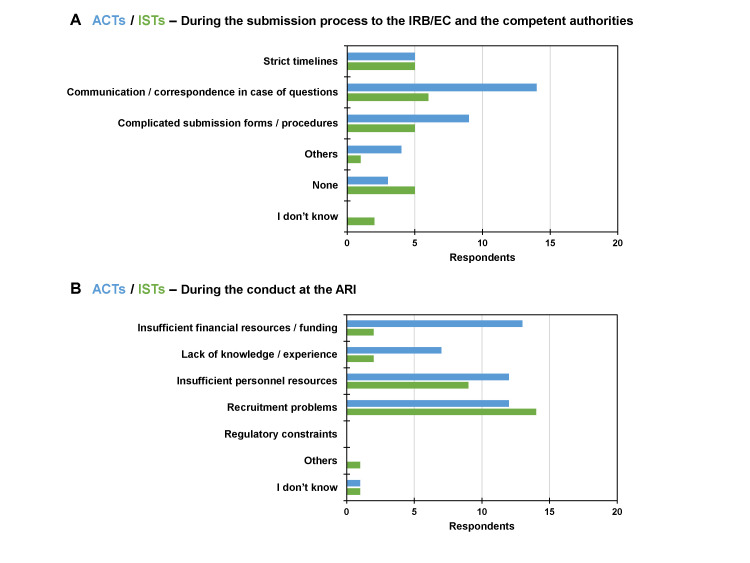
Challenges for ACTs and ISTs. Multiple answers from respondents were possible. **Panel A.** During the submission process to the IRB/EC and the competent authorities. **Panel B.** During the conduct at the ARI. ACT – academic clinical trial, ARI – academic research institution, IRB/EC – institutional review boards/ethics committees, IST – industry-sponsored trial.

For ACTs vs. ISTs, the main challenges during the submission process to the institutional review board (IRB) or ethics committee (EC) and the competent authorities were, according to the respondents, ‘communication/correspondence in case of questions’ (70% vs. 33%), ‘complicated submission forms/procedures’ (45% vs. 28%), ‘strict timelines’ (25% vs. 28%), and ‘others’ (20% vs. 6%, i.e. lack of awareness of procedures, unclear regulatory requirements, lack of knowledge especially in case of studies that are not under the law of the Medicinal Products Act in Germany, local ethics committee having no strict timelines for review and tending to need several months for ACTs vs. lack of knowledge of contract research organisation personnel for ISTs, respectively) (**Figure 4**, Panel A). Three respondents (15%) for ACTs and five (28%) for ISTs replied that there were no hurdles. Two respondents (11%) did not know about any hurdles for ISTs.

During the conduct of clinical trials at the ARI, the reported hurdles for ACTs vs. ICSTs were ‘insufficient financial resources/funding’ (65% vs. 11%), ‘insufficient personnel resources’ (60% vs. 50%), ‘recruitment problems’ (60% vs. 78%), and ‘lack of knowledge/experience’ (35% vs. 11%) (**Figure 4**, Panel B). One respondent did not know in each case.

We compared the difference in reported challenges for ACTs vs. ISTs using Fisher exact test and found no significant difference between the answers provided for ACTs vs. ISTs for either challenges during the submission process to the IRB/EC and the competent authorities (*P* = 0.272) or for challenges during the conduct at the ARI (*P* = 0.06).

**Figure 5:**
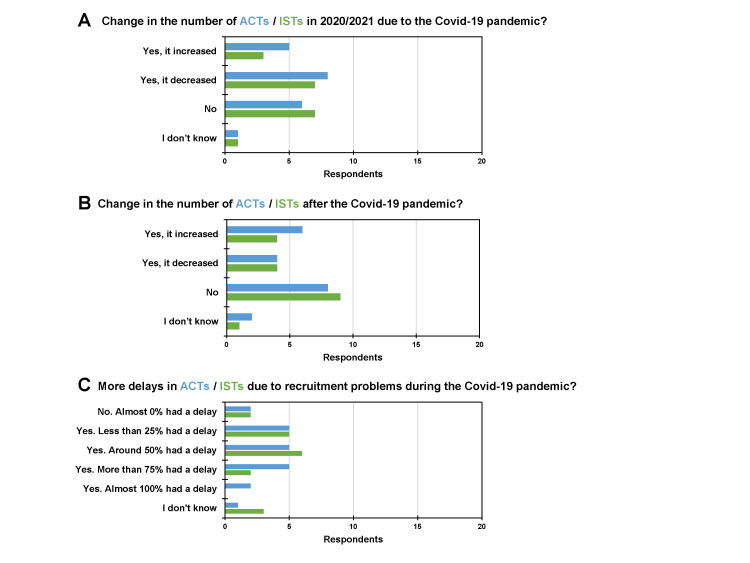
Impact of the COVID-19 pandemic on ACTs and ISTs. **Panel A.** Change in the number of trials in 2020/2021 due to the COVID-19 pandemic. **Panel B.** Change in the number of trials after the COVID-19 pandemic. **Panel C.** Delays due to recruitment problems during the COVID-19 pandemic. ACT – academic clinical trial, IST – industry-sponsored trial.

We analysed the impact of the coronavirus disease 2019 (COVID-19) pandemic on ACTs and ISTs (**Figure 5**). The responses were heterogeneous for ACTs vs. ISTs; the number of clinical trials in 2020/2021 either increased (25% vs. 17%), decreased (40% vs. 39%), or did not change (30% vs. 39%) (**Figure 5**, Panel A). Similarly, after the COVID-19 pandemic, the number of trials either increased (30% vs. 22%), decreased (20% vs. 22%), or did not change (40% vs. 50%) (**Figure 5**, Panel B). Seven respondents (35%) indicated that more than 75% of ACTs experienced a delay due to recruitment problems during the COVID-19 pandemic (**Figure 5**, Panel C). In contrast, two out of 18 participants (11%) reported such a delay for ISTs. We did not see any significant difference between ACTs and ISTs, as per Fisher exact test (*P* > 0.05).

## DISCUSSION

According to our respondents, ACTs predominantly rely on national, industrial, and institutional grants. It is important to distinguish ACTs from ISTs; although the former may receive industrial grants, they remain under academic sponsorship, while the latter are managed entirely by the industry. Martin et al. [13] reported that the median cost of conducting a study from protocol approval to final clinical trial report was USD 3.4 million for phase I trials involving patients, USD 8.6 million for phase II trials, and USD 21.4 million for phase III trials. For phase III trials, personnel expenses account for 37%, and outsourcing for 22% of the total trial costs [13]. Despite these figures, 35% of our participants indicated that 50% or more of ACTs faced significant funding deficiencies.

### Challenges in knowledge and experience

A notable challenge for ACTs compared to ISTs is the lack of knowledge and experience. Despite this, only 25% reported that academic sponsors reach out to ARIs most of the time for advice during the planning phase of ACTs. Complicated submission procedures also pose significant obstacles. According to ICH GCP guidelines, sponsors are responsible for the successful execution of clinical trials and must ensure adequate training and financial resources. Our survey highlights that academic researchers often struggle with these requirements. Simultaneously, academic projects are crucial for the treatment of patients as they are typically not profit-oriented, suggesting that regulatory requirements should be consistent between ACTs and ISTs to ensure high ethical and scientific standards. Academic sponsors should more frequently use the expertise of ARIs to support them throughout the clinical trial process.

### Budgetary constraints and impact

ACTs are generally conducted by small teams and often face financial constraints due to their reliance on external funding. Researchers eager to commence trials might start without securing the full budget, leading to issues such as delays in recruitment and higher study costs. Through shared infrastructures, for instance, redundancies between research groups and ARI personnel can be reduced, thus saving financial resources.

McLennan et al. highlighted that budget estimations for ACTs in Switzerland are frequently inaccurate due to poor planning and unrealistic expectations [1]. Delays in recruitment might also increase study costs, as described by Speich et al. in the Prednisone-Trial [14]. Chronic underfunding of ACTs presents a significant problem, as ACTs are vital for addressing patient-centred research questions that may not attract substantial industry interest [4,15]. The limited resources for ACTs contribute to a higher incidence of premature trial discontinuation compared to ISTs [16], raising ethical concerns and impacting participants, researchers, and the scientific community. This not only weakens trust in the research enterprise and sponsor organisations, but also wastes resources, such as financial investments, time, and effort that could have been directed towards more promising research initiatives [17].

### Case study – funding challenges

Our experience with an academic phase-one study in infectious diseases illustrates the impact of funding challenges where, despite securing multiple grants from non-profit organisations, the sponsor faced issues. Since they had to cover all costs from medical writing, submissions, study setup, and study conduct, the budget was calculated very precisely. One of the study drugs had a delivery difficulty and prolonged trial duration. Moreover, participant recruitment took longer than planned due to a high number of pre-screening failures. The study protocol needed to be changed during the study, leading to additional costs. Dropout rates of study participants were also higher than initially expected. Ultimately, this led to higher study costs. Our ARI had to reduce its budget to support the ACT until completion and the sponsor needed to apply for new grants. For academia, such financial setbacks can, at worst, lead to early study termination, resulting in a complete waste of resources and posing significant ethical issues for participants already involved.

### Recruitment challenges

Recruitment issues are prevalent in both ACTs and ISTs. Our survey participants found that 60% of ACTs and 78% of ISTs encounter recruitment problems. Competing studies and strict inclusion and exclusion criteria can hinder recruitment efforts. The COVID-19 pandemic intensified these delays, and both academic and commercial clinical trials temporarily halted the recruitment of new participants to guarantee participant safety [18,19]. Further challenges for ACTs include continuous oversight, training of study personnel, expertise in statistics, data management, and medical writing [4], as well as obtaining placebos for randomised controlled trials [20].

### A closer look at ISTs

Industrial studies benefit from substantial budgets. Each study contributes to a larger study portfolio that depends on the expertise of marketing professionals, scientific experts, and management staff to increase shareholder value. Pharmaceutical companies have optimised monitoring and reporting processes, ensuring comprehensive data collection, with the entire process facilitated by a contract research organisation [21]. Speich et al. [3] reported that out of 326 randomised controlled trial protocols approved in 2012, only 16% (n/N = 23/147) of ACTs reported their results in a trial registry, in contrast to 84% (n/N = 150/179) of ISTs. However, ISTs face concerns regarding scientific integrity due to their typically large budgets and commercial drive. There is an ongoing discussion, both within the research community and among the public, about potential conflicts of interest that may arise when clinical research funding comes from organisations with a vested interest in the outcome [22,23]. Amongst others, further examples potentially raising integrity concerns of ISTs include data collection and analysis methods, as sponsors might prioritise results aligning with their financial interests. Additionally, publication bias is a concern, as ISTs may selectively publish positive results, potentially distorting the overall evidence base. Financial ties between industry sponsors and researchers can also generate conflicts of interest, casting doubt on the independence and objectivity of study findings [23]. It therefore requires transparency in disclosing funding sources, trial protocols, and conflicts of interest, as well as rigorous adherence to ethical guidelines and regulatory standards. Independent oversight and peer review also help safeguard the scientific integrity of ISTs, ensuring that they contribute valid and reliable evidence to the medical community.

### Role of ARIs

ARIs play a critical role in supporting academic researchers [24,25], with our survey indicating that 55% of them also act as sponsors. With their knowledge and experience concerning regulatory affairs, data management, and quality management, ARIs are suitable for this task. They provide essential services and support to external sponsors, namely quality management, monitoring, project management, regulatory and legal affairs, education and training, data management, vigilance, biostatistics, contract management, research consultation, ethics affairs, protocol development, budget management, feasibility assessment, payment management, publication of trial results, IT-support/development, medical writing, training or providing study nurses who are and are not in contact with patients, archiving, and technology transfer [26]. Offering quality management services and expertise in regulatory and legal affairs was considered most important for emerging ARIs. Moreover, education and training services ensure that clinicians are well-trained on GCP and legislation. Finally, ARIs should evaluate whether they have the expertise and resources available to offer operative services such as study conduct. All ARIs within our survey applied the ICH GCP guidelines to guarantee ethical and high-quality clinical research, which is why we observed no geographical differences related to the quality of performed research. By reducing redundancies and optimising resource use, ARIs can enhance the efficiency of ACTs. Digital tools and technologies are currently being developed to further support clinical trial management. Low-cost products or free-to-use software can help mostly ACTs achieve better trial management capacities for example during recruitment of trial participants [27]. As they are sponsored by the pharmaceutical industry, ISTs often have their private software for a better clinical trial overview. These applications help both participants and researchers in coordinating their activities, ultimately leading to faster termination and publication of clinical trial results. Financial support for academic research varies between countries. According to data from the World Bank website, research and development expenditure reached 3.36% of GDP in Switzerland, 3.14% in Germany, 2.43% in China, 1.45% in Italy, 1.21% in Thailand and 1.40% in Turkey (most recent data for the year 2021) and 1.83% in Australia and 0.28% in Nigeria (most recent data for the year 2019) [28].

### Impact of ACTs

Importantly, ACTs cover key areas of medical research, often going beyond the scope of ISTs by generating data on the effectiveness and safety of drugs in the real-world setting (phase IV clinical trials) and promoting evidence-based medicine. Lathyris et al. [29] reported that out of 577 randomised trials involving the 15 companies that had sponsored the largest number of registered trials in ClinicalTrials.gov in 2006, 82% had a single industry sponsor. The compared interventions belonged to a single company in 67% of the trials and only 18 trials assessed a head-to-head comparison of different active interventions developed by different companies [29]. According to Blümle et al. [2], results of ACTs were more often published as a journal article while results of ISTs were more often published in study registries. Moreover, international ISTs less often gained impact by inclusion in systematic reviews or guidelines than ACTs [30]. Collaboration between academics and industry in clinical trials is common in the development of drugs, vaccines and devices. According to Rasmussen et al. [31], academics evaluated this cooperation (in the design, conduct, and reporting of the clinical trials) as beneficial, even though some reported a loss of academic freedom and disagreements, mostly concerning trial design and reporting.

### Limitations

The survey implemented here was a self-developed instrument and reliance on self-reported data from a small sample size may introduce bias. While the participation of 20 respondents from 15 countries is satisfactory, the findings may not be generalisable to regions not represented, such as North and South America. Since the USA is a key market for clinical research and drug applications, their opinions would have been highly valuable. Future studies should explore the challenges faced by ARIs outside ICN as well as private research institutions and contract research organisations to provide a broader perspective.

## CONCLUSIONS

Our findings suggest that ISTs generally face fewer funding issues and communication challenges with authorities or ethics committees compared to ACTs. To address these issues, ARIs should be created to support and advise academic researchers on all aspects, including project planning and implementation, with a particular focus on feasibility and funding. Through concentrated knowledge and shared infrastructures, redundancies can be reduced, and financial and human resources can be used optimally. In this sense, we outline five important action points for sponsors of ACTs in **Table 2**. Intensified collaboration between academic sponsors and ARIs will streamline clinical trial processes, guide the study personnel, and enhance the overall success of clinical trials, benefitting both researchers as well as patients.

**Table 2:** Five action points for sponsors of ACTs

Action point	Description
Contact/seek general support from local ARI	Engage with ARIs for comprehensive support.
Budget advice by ARI	Obtain financial planning and budgeting assistance.
Submissions to IRB/EC and competent authorities by ARI	Use ARIs for regulatory submissions and communications.
Support with project management tasks by ARI	Leverage ARIs for managing project tasks and coordination.
Regular GCP training by ARI	Ensure ongoing GCP training and qualification of academic researchers.

ACT – academic clinical trial, ARI – academic research institution, GCP – Good Clinical Practice, IRB/EC – institutional review boards/ethics committees

## Additional material


Online Supplementary Document
