# Supplementary material for: Academic vs. industry-sponsored trials: A global survey on differences, similarities, and future improvements
Source: J Glob Health. 2024 Nov 22;14:04204. doi: 10.7189/jogh.14.04204 (PMC11583285; doi:10.7189/jogh.14.04204)
Supplement: Online Supplementary Document [file jogh-14-04204-s001.pdf]

ICN International Clinical Trial Center Network

ICN SURVEY

ACADEMIC CLINICAL TRIALS

INTRODUCTION:

Dear ICN Member,

We invite you to answer this questionnaire concerning academic clinical trials and hope to learn more about each institution and to gain insights into the different processes and regulatory frameworks. We plan to share the findings within the ICN and publish the results. We will mention each contributor of the ICN Study Group in a supplement. Your participation is entirely voluntary. The answers that you give will be kept confidential and will be used only for research purposes. Please ensure that the participants of the survey have access to Management Review in your institution in order to answer the questions accurately.

The questionnaire will take between 30-40 minutes. Below each question we've added a comment section. This field is optional. You are more than welcome to share more detailed information or your personal experience / opinion in the comment section of each question.

GENERAL DATA

1. Date

2. Please choose your country / jurisdiction

Australia
Austria
China
Germany
Hong Kong
Hungary
Israel
Italy
Japan
Nigeria
Russia
Singapore
Sweden
Switzerland
Taiwan
Thailand
Turkey
Uganda
United Kingdom
USA
Other, please specify

3. Please enter the name of your institution:

4. Please select any topic your institution is involved in: *(more than one answer possible)*

a. Phase I clinical trials (First in Human trials, PK/PD trials)
b. Phase II clinical trials (Exploratory trials)
c. Phase III clinical trials (Confirmatory trials)
d. Phase IV clinical trials (Post-market trials)
e. Academic clinical trials / Investigator initiated trials (IITs)
f. Industry sponsored trials
g. Non-clinical trials
h. Focus on specific medical specialties (e.g. Oncology, Cardiology,…) Please specify if applicable: ____

5. Please specify your function and/or department:

☐ Management / Head	☐ Quality Manager	☐ Monitor	☐ Study Coordinator / Study Nurse	☐ Principal Investigator	☐ Investigator
☐ Data Manager	☐ Ethics Committee	☐ Data Protection Officer	☐ Regulatory	☐ Legal department	☐ Project Manager
☐ Pharmacovigilance	☐ Budgeting Services	☐ Sponsor Oversight	☐ Other, please specify:______		

If you agree, please enter your name and e-mail address so we can send you updates of this project and contact you in case we need further information:

a. First name: ______________________________
b. Last name: _______________________________
c. E-mail address: ___________________________

ACADEMIC CLINICAL TRIALS

In the following survey, we would like to find out more about the regulatory framework for conducting academic clinical trials, also called investigator initiated trials (IITs) or studies (IIS), in your country / jurisdiction.

As stated by Arun Bhatt [New clinical trial rules: Academic trials and tribulations. Perspect Clin Res 2019;10:103-5], “Academic trials or investigator-initiated studies (IIS) are clinical studies conceived, planned, and managed by individual physician–researchers or an institution or a group of collaborative clinical researchers or institutions.” The investigator assumes the sponsor role, thus being responsible for the conducted academic/industry-independent research as sponsor investigator. Funding is assured through grants, institutional funds, governmental funds, etc. In contrast, for industry sponsored trials the industry keeps the overall responsibility as sponsor.

ICH GCP definition of “Sponsor”: An individual, company, institution, or organization which takes responsibility for the initiation, management, and/or financing of a clinical trial.

ICH GCP definition of “Investigator”: A person responsible for the conduct of the clinical trial at a trial site. If a trial is conducted by a team of individuals at a trial site, the investigator is the responsible leader of the team and may be called the principal investigator.

Specifications for the following questionnaire:

- By “conducting” a clinical trial we mean the sum of operational activities related to a clinical trial such as planning, recruiting and analysis
- By “consulting” a clinical trial we mean the passive input such as giving advice to researchers, i.e. no operational activities during a clinical trial

Regulatory framework for clinical trials

1. Does the ICH-GCP guideline apply for clinical trials in your country/jurisdiction?
    a. Yes
    b. No
    c. I don't know

Additional information (optional):

2. If *1a*: Is the ICH-GCP guideline linked to local / national legal text or regulation/guideline?
    a. Yes
    b. No
    c. I don't know

Additional information (optional):

3. Does your country / jurisdiction have a specific national law for clinical trials?
    a. Yes
    b. No
    c. I don't know

Additional information (optional):

4. Do other local or institutional standards / guidelines apply to clinical trials?
    a. Yes: Please specify: ____________________
    b. No
    c. I don't know

Additional information (optional):

General information about academic clinical trials

5. Is the definition of academic clinical trials in your country / jurisdiction the same as the one given in the introduction by Arun Bhatt i.e. “Academic trials or investigator-initiated studies (IIS) are clinical studies conceived, planned, and managed by individual physician–researchers or an institution or a group of collaborative clinical researchers or institutions”?
    a. Yes
    b. No: Please specify: ____
    c. I don’t know

Additional information (optional):

6. How is your institution involved in academic clinical trials? *(more than one answer possible)*
    a. My institution acts as sponsor.
    b. My institution conducts academic clinical trials.
    c. My institution consults academic clinical trials.
    d. My institution is not involved in any academic clinical trials. *(if answer d is checked, please go directly to question 17 “General Information about industry sponsored trials”, page 13)*
    e. Others: Please specify: _________
    f. I don’t know

Additional information (optional):

7. If *6a, 6b or 6c*: How high was the share of academic clinical trials in the services provided by your institution (consulting, planning, conduct/recruiting, analysis, etc.) in 2021? Please provide a rough estimate.
    a. Almost 0%
    b. Less than 25%
    c. Around 50%
    d. More than 75%
    e. Almost 100%

f. I don’t know

Additional information (optional):

Funding of academic clinical trials

8. Which are the main funding sources for academic clinical trials that your institution conducted and/or consulted? *(more than one answer possible)*
    a. Institutional grants/funds
    b. National grants/funds
    c. International grants/funds
    d. Private / philanthropic funds (e.g. foundations)
    e. Industry / private grant provider
    f. Others: Please specify: _________
    g. I don't know

Additional information (optional):

9. In 2021, how many academic clinical trials that your institution conducted and/or consulted were insufficiently funded? Please provide a rough estimate.
    a. Almost 0%
    b. Less than 25%
    c. Around 50%
    d. More than 75%
    e. Almost 100%
    f. I don't know

Additional information (optional):

Submission process for academic clinical trials

10. Do sponsors/sponsor-investigators of academic clinical trials in your country / jurisdiction reach out to the local clinical trial units (CTUs) for advice in the planning phase?
    Always / Most of the Time / Sometimes / Rarely / Never / I don't know

Additional information (optional):

11. What are the main hurdles that academic clinical trials faced during the submission process to the Institutional Review Board (IRB) / Ethics Committee (EC) and the competent authorities? *(more than one answer possible)*
    a. Strict timelines
    b. Communication/correspondence in case of questions
    c. Complicated submission forms / procedures
    d. Others: Please specify: _________
    e. None
    f. I don't know

Additional information (optional):

Conduct of academic clinical trials

12. In 2021, most academic clinical trials at your institution were…
    a. National monocentric clinical trials
    b. National multicentric clinical trials
    c. International clinical trials
    d. I don't know

Additional information (optional):

13. What are the main hurdles during the conduct of academic clinical trials at your institution? *(more than one answer possible)*
    a. Insufficient financial resources / funding
    b. Lack of knowledge / experience
    c. Insufficient personnel resources
    d. Recruitment problems
    e. Regulatory constraints
    f. Others: Please specify: ____________
    g. I don't know

Additional information (optional):

Impact of the Covid-19 pandemic on academic clinical trials

14. Did the number of academic clinical trials that your institution conducted and/or consulted change in 2020/2021 due to the Covid-19 pandemic compared to pre-Covid years (e.g. in 2019)?
    a. Yes, it increased
    b. Yes, it decreased
    c. No
    d. I don't know

Additional information (optional):

15. Did the number of academic clinical trials that your institution conducted and/or consulted change AFTER the Covid-19 pandemic, i.e. in the first half of 2022 (from January until June), compared to the first half of 2021?
    a. Yes, it increased
    b. Yes, it decreased
    c. No
    d. I don't know

Additional information (optional):

16. Did academic clinical trials experience more delays due to recruitment problems during the Covid-19 pandemic (i.e. in 2021) compared to pre-Covid years (e.g. in 2019)?
    a. Yes: Please estimate the percentage of academic clinical trials with a delay: Almost 0% Less than 25%, Around 50%, More than 75%, Almost 100%
    b. No
    c. I don't know

Additional information (optional):

General information about industry sponsored trials

17. How is your institution involved in industry sponsored trials? *(more than one answer possible)*
    a. My institution conducts industry sponsored trials.
    b. My institution consults industry sponsored trials.
    c. My institution is not involved in any industry sponsored trials. *(if answer c is checked, please go directly to question 25 "Ethical and strategical aspects of academic clinical trials", page 17)*
    d. Others: Please specify: _________
    e. I don't know

Additional information (optional):

18. If *17a or 17b*: How high was the share of industry sponsored trials in the services provided by your institution (consulting, planning, conduct/recruiting, analysis, etc.) in 2021? Please provide a rough estimate.
    a. Almost 0%
    b. Less than 25%
    c. Around 50%
    d. More than 75%
    e. Almost 100%
    f. I don't know

Additional information (optional):

Submission process for industry sponsored trials

19. What are the main hurdles that industry sponsored trials faced during the submission process to the Institutional Review Board (IRB) / Ethics Committee (EC) and the competent authorities? *(more than one answer possible)*
    a. Strict timelines
    b. Communication/correspondence in case of questions
    c. Complicated submission forms / procedures
    d. Others: Please specify: _________
    e. None
    f. I don't know

Additional information (optional):

Conduct of industry sponsored trials

20. In 2021, most <u>industry sponsored trial</u>s at your institution were…
    a. National monocentric clinical trials
    b. National multicentric clinical trials
    c. International clinical trials
    d. I don't know

Additional information (optional):

21. What are the main hurdles during the conduct of <u>industry sponsored trials at your institution</u>? *(more than one answer possible)*
    a. Insufficient financial resources / funding
    b. Lack of knowledge / experience
    c. Insufficient personnel resources
    d. Recruitment problems
    e. Regulatory constraints
    f. Others: Please specify: ____________
    g. I don't know

Additional information (optional):

Impact of the Covid-19 pandemic on industry sponsored trials

22. Did the number of industry sponsored trials that your institution conducted and/or consulted change in 2020/2021 due to the Covid-19 pandemic compared to pre-Covid years (e.g. in 2019)?
    a. Yes, it increased
    b. Yes it decreased
    c. No
    d. I don't know

Additional information (optional):

23. Did the number of industry sponsored trials that your institution conducted and/or consulted change AFTER the Covid-19 pandemic, i.e. in the first half of 2022 (from January until June) compared to the first half of 2021?
    a. Yes, it increased
    b. Yes, it decreased
    c. No
    d. I don't know

Additional information (optional):

24. Did industry sponsored trials experience more delays due to recruitment problems during the Covid-19 pandemic (i.e. in 2021) compared to pre-Covid years (e.g. in 2019)?
    a. Yes: Please estimate the percentage of industry sponsored trials with a delay: Almost 0%, Less than 25%, Around 50%, More than 75%, Almost 100%
    b. No
    c. I don't know

Additional information (optional):

Ethical and strategical aspects of academic clinical trials

25. What are in your opinion the main advantages/reasons of academic clinical trials? *(more than one answer possible)*
    a. Independence from industry
    b. Patient-centered trials / Answering questions relevant to patient care
    c. Publication in high ranking journals
    d. Others: Please specify: _______________
    e. I don't know

Additional information (optional):

26. What are in your opinion the greatest disadvantages/risks of academic clinical trials? *(more than one answer possible)*
    a. Lack of financial resources
    b. Lack of personnel resources / experiences
    c. Publication-driven motivations
    d. Others: Please specify:_______________
    e. I don't know

Additional information (optional):

27. Are all academic clinical trials that your institution conducts and/or consults registered in a clinical trial registry and updated regularly?
    a. Yes. Please specify the name of the registries you use: __________________
    b. No: Please specify: ____
    c. I don't know

Additional information (optional):

28. During the design of an academic clinical trial, how often do you focus on patient and public involvement, e.g. through patient representatives? Please provide a rough estimate.
    a. Almost 0%
    b. Less than 25%
    c. Around 50%
    d. More than 75%
    e. Almost 100%
    f. I don’t know

Additional information (optional):

29. Concerning the ethical issue of storage and further use of patient data and samples, do you think patients understand sufficiently the biobanking process after informed consent (e.g. where the samples are stored, who has access to the samples, etc.)?
    a. Yes
    b. No
    c. I don’t know

Additional information (optional):

Greenhouse gas emissions / environmental impact of clinical trials

Climate change is a major current threat to both our planet and humankind. All economic sectors need to reduce their greenhouse gas emissions in order to limit global temperature increase. We therefore intend to gather more information about how your institution and your country/jurisdiction contribute to reduce greenhouse gas emissions for clinical trials.

30. During submission of a clinical trial to the IRB / EC or the competent authorities, is there a focus on the carbon footprint of each trial in your institution? *(more than one answer possible)*
    a. Yes, greenhouse gas emissions of a clinical trial are assessed
    b. No, greenhouse gas emissions are not assessed
    c. The IRB / EC advise researchers on reducing the carbon footprint of their clinical trials
    d. The competent authorities advise researchers on reducing the carbon footprint of their clinical trials
    e. I don't know

Additional information (optional):

31. How many percent of the total number of <u>academic clinical trials</u> in your institution focus on limiting their greenhouse gas emissions? Please provide a rough estimate.
    a. Almost 0%
    b. Less than 25%
    c. Around 50%
    d. More than 75%
    e. Almost 100%
    f. I don't know

Additional information (optional):

32. How many percent of the total number of <u>industry sponsored trials</u> in your institution focus on limiting their greenhouse gas emissions? Please provide a rough estimate.
    a. Almost 0%
    b. Less than 25%
    c. Around 50%

d. More than 75%
e. Almost 100%
f. I don't know

Additional information (optional):

33. Which healthcare sector activities do you believe contribute the most to greenhouse gas emissions? **Please select three answers**.
    a. Operational/Institutional fuel use
    b. Transport & distribution
    c. Electricity
    d. Travel / Employee commuting
    e. Agriculture/Food
    f. Waste treatment
    g. End-of-life treatment of sold products
    h. Others? Please specify:_______________
    i. I don't know

Additional information (optional):

34. Which measures has **your institution** taken to reduce greenhouse gas emissions? *(more than one answer possible)*

    a. Assessing the carbon footprint of your institution (e.g. using available online calculators)
    b. Using renewable energy sources
    c. Minimizing travelling
    d. Preference of teleconferencing and/or videoconferencing
    e. Others? Please specify: _________
    f. None
    g. I don't know

Additional information (optional):

35. Which measures are being implemented in **your country/jurisdiction** to reduce the greenhouse gas emissions of clinical trials? *(more than one answer possible)*

    a. Assessing the carbon footprint of clinical trials (e.g. using available online calculators)
    b. Using renewable energy sources
    c. Minimizing travelling
    d. Preference of teleconferencing and/or videoconferencing
    e. Managing health care waste
    f. Assessing the carbon footprint of clinical trials in grant applications
    g. Others: Please specify: __________
    h. None
    i. I don't know

Additional information (optional):

END

Thank you very much for completing the survey!
If you have any questions or comments, please contact jean-marc.hoffmann@usz.ch
